# Clinical benefit and improvement of activity level after reconstruction surgery of Charcot feet using external fixation: 24-months results of 292 feet

**DOI:** 10.1186/1471-2474-15-392

**Published:** 2014-11-22

**Authors:** Ulrich Illgner, Tymo Budny, Inna Frohne, Nani Osada, Jan Siewe, Hans H Wetz

**Affiliations:** Clinic for Technical Orthopedic Surgery, University Hospital of Muenster, Albert-Schweitzer-Campus 1, Muenster, Germany; Institut for clinical mathematics, University Hospital of Muenster, Muenster, Germany; Clinic for Orthopedic Surgery and Traumatology, University Hospital of Cologne, Cologne, Germany

**Keywords:** Charcot feet, Charcot neuropathy, Reconstructive surgery, Diabetes mellitus, External fixator

## Abstract

**Background:**

Reconstruction of Charcot feet remains a surgical challenge. The goal of this study was to investigate safety and clinical benefit from reconstruction of Charcot feet using an external fixator. There is limited valid data regarding long-term outcomes for Charcot foot procedures.

**Methods:**

In a retrospective study, 292 Charcot feet (282 patients) undergoing reconstructive procedures in our clinic from 1996–2010 were included (93 female, 189 male, mean age 57.9 years). Average follow-up was 24.1 months. Exclusion criteria were previous major amputation on the same side. All patients underwent surgery using a Hoffmann II external fixator for six to eight weeks with offloading. The fixator was then removed, and a customized AFO with full weight bearing was applied for another 11 months. After one year, patients received customized orthopedic shoes.

**Results:**

Initial amputations were avoided. Patient activity improved significantly by more than 1 level (SD 0.67, p < .001) according to the Hoffer activity score for lower limb amputees. The most common minor complication was persistent or recurrent ulceration in 67 feet (23%). Secondary amputation (after failure of external fixation) was required in only 12 patients (6.2%). Orthopedic shoes were used by approximately 34% of patients 18 months after surgery.

**Conclusions:**

Reconstructive surgery of Charcot feet using external fixation is a safe and economically feasible procedure. Activity levels improved significantly by more than 1 level (p < 0.01), severe complications were rare, and secondary amputation was required in only 12 patients (6.2%) of a high-risk patient population. Use of an external fixator offers the advantage that all extraneous material is removed after six weeks; thus, there is no risk of broken screws or plates and the associated potential complications.

**Electronic supplementary material:**

The online version of this article (doi:10.1186/1471-2474-15-392) contains supplementary material, which is available to authorized users.

## Background

Published descriptions of neuropathic arthropathy by Charcot initially appeared in 1868[1]. Different types of polyneuropathy are central to its development[2]. In recent studies, an infectious etiology for the development of Charcot neuropathy (CN) has been proposed[3]. Although diabetes mellitus is the most common cause for CN in western and developing countries, there are many other etiologies for its development, e.g. alcohol abuse, rheumatoid arthritis, toxicity (e.g. induced by methotrexate), idiopathic, or as a result of leprosy or other diseases[4]. “The interaction of several component factors (diabetes, sensory-motor neuropathy, autonomic neuropathy, trauma, and metabolic abnormalities of bone) results in an acute localized inflammatory condition that may lead to varying degrees and patterns of bone destruction, subluxation, dislocation, and deformity “(International consensus report[2]). The number of patients with diabetes mellitus is rising in western and developing countries, most likely with many undiagnosed patients[5]. The WHO states 347 million patients with diabetes mellitus worldwide (WHO fact sheet no 312, updated Nov 2014[6]).

The treatment and reconstruction of Charcot feet remains a surgical challenge. Most cases exhibit atypical, multidirectional destruction and instability, often combined with major bone loss and ulceration with infection[7]. In addition, most patients have comorbidities and suffer from diabetes mellitus, nephropathy, and/or peripheral vascular disease. Longer operative duration, hemoglobin A1c levels >7 mg/dl, and peripheral neuropathy elevate complication rates, especially regarding wound healing and infections[8, 9]. Infections with P. aeruginosa in particular have been associated with poor outcomes[7].

Various surgical approaches using internal, external, or combined fixation techniques have been published. However, valid long-term outcome studies with sufficient patient numbers are lacking[10, 11]. In a case report, Facaros combined internal plate fixation with a ring external fixator (i.e. Ilizarov)[12]. Grant reported the long-term outcomes of 46 patients using the SF-36 questionnaire. These patients underwent various types of surgery using either column beaming or external fixation, with or without bone grafting. Amputation was required in 4% of cases, and osteomyelitis occurred in 26%, but still, “none of the demographic variables was statistically significantly associated with patient outcomes as measured by the SF-36 and the patient satisfaction survey”[11].

It is important to investigate outcome markers relevant to patients. Ambulation and activity level are at first and most important impaired when ulcers develop on the foot. Usually these ulcers are located plantar at the sites of most load bearing and most affection of CN. Offloading and/or reconstruction surgery are standard treatment options meaning that these patients cannot walk at all or only in very limited matter. Because of the mostly bilateral polyneuropathy and in most cases high elevated BMI, many patients are not able to walk on crutches. There is a high risk to become depend on foreign help and wheelchairs. Social life seems nearly impossible and consecutive complications (e.g. thrombosis, pneumonia) can follow. Once an ulcer is healing there is a high rate of recurrence and the whole problems restarts.

Charcot neuroarthropathy (CN) is a lifelong condition with high rates of recurrence, and broken screws or plates in particular can cause future ulceration. Amputation rates, ability to walk (outside), need for orthotic devices, and recurrence of ulceration and/or infection are the benchmarks for a successful outcome in our opinion. Radiological results and fusion rates are also important for evaluation, but they should not be considered outcome measures.

The goal of our study was to find out, if our proposed treatment regimen with full weight bearing as early as 6 weeks after surgery leads to an improvement of activity level and amputations can be kept at a low level.

## Methods

From 1996–2010, 292 feet (282 patients) were included in this study (Table 1). Average follow-up was 24.1 months (3–49 months). 288 patients underwent standard procedures in that time, but six patients were excluded because of incomplete reports. Medical records, including pre and post-operative radiographs and information regarding walking ability and use of orthotic devices, were examined retrospectively in the year 2011 by the examination doctor inspecting the worn orthotic devices or shoes and the walking ability of the patients.

**Table 1:** **A, B: Patient’s data and secondary diagnoses**

Patients (n=)	282
Foot surgeries (n=)	292
Age (y)	57,9 (SD 10.16)
Male	189 (77.1%)
Female	93 (32.9%)
BMI	32,8 (19.9 -59.7, SD 6.9)
Diabetes type 1	37 (13.1%)
Diabetes type 2	207 (73.4%)
No Diabetes	38 (13.4%)
Polyneuropathy	282 (100%)
Hypertension	182 (64.5%)
Retinopathy	111 (39.4%)
Nephropathy	90 (31.9%)
Coronary heart disease	54 (19.1%)
Peripheral vessel disease	53 (18.8%)
Alcohol abuse	9
Rheumatoid arthritis	9
Hereditary motosensoric neuropathy	2
M. von Recklinghausen	1
Idiopathic	17
Total	38

Activity level was measured during the controls using the Hoffer score, which consists of 4 levels, activity level 0 means “non walker”, level 1 means “therapy walker”, level 2 “household walker” and level 3 “community walker”.

Exclusion criteria were arthrodesis of reconstructive foot surgery without Charcot neuroarthropathy (CN), and major amputation on the same side.

All patients gave formal consent to participate in this study and all demands of the declaration of Helsinki were fulfilled. The ethic committee of the University of Muenster approved the study.

Surgery was performed in all stages of Eichenholtz, most patients were in Eichenholtz stage II. We do not see Eichenholtz stage I as a contra indication for surgery using an external fixator. Indications for surgery included instability of the tibio-calcaneal, talo-calcaneal (subtalar), Chopart’s, or Lisfranc’s joints eligible for stabilization in a total contact cast or AFO, exostoses causing skin ulceration, therapy-resistant ulceration, and/or osteomyelitis. With the use of external fixation, reduction and stable immobilization are possible without direct contact to the affected area. When deformity was minimal and the tibio-calcaneal joint was not affected (Sanders I-III), we used Steinmann pins for reduction. The pins were regularly removed after 6 weeks. Only is case of infection pins were removed earlier and we did not let the pins stay in longer. Patients wore an AFO with full weight bearing for a minimum of 12 months. With an AFO, patients are able to return to work. Our goal for the patients was the ability to walk in customized orthopedic shoes after 12 months.

**Figure 1:**
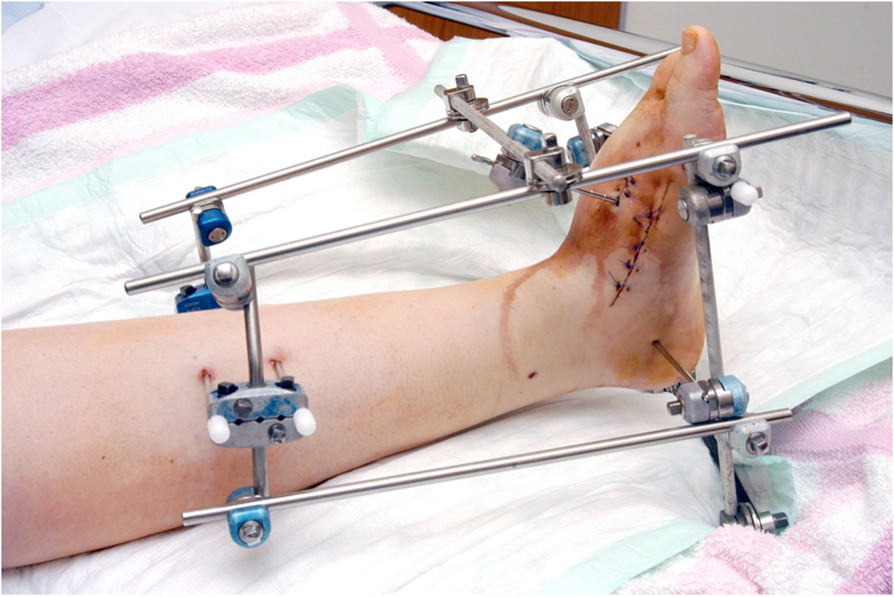
**Reduction using a double**-**frame external fixator.**

When major edema and ulcerations were visible, the joint was immobilized preoperatively in a cast. Systemic (I.V.) antibiotics were given for major infections as soon as possible according to culture and sensitivity tests.The operation starts with a tenotomy of the Achilles tendon. Then we approached from the convex side of the affected part of the foot. In severe deformities we performed a bilaterally approach. Then debridement and resection of infected ligaments, capsules, and/or exostoses followed. Unaffected cancellous bone was saved and used as autologous graft. Local antibiotics (Septopal® chains) were used in cases of macroscopic infection. Steinmann pins with a central thread were passed through the metatarsal heads, then into the calcaneus and tibia and were stabilized by an external double frame Hoffmann-II-Fixator (Figure 1).

In 265 cases, Hoffmann II external fixators, and in 17 patients, Steinmann pins were used for reduction. In seven cases, reduction was performed after resection of a metatarsal wedge. A cast for the lower limb was then applied for 6 weeks. The pins were removed after 6 weeks, and patients were mobilized with full weightbearing in an interim AFO for a minimum of 12 months once swelling was decreased. AFOs were made from carbon fibers, and after an average 3–4 months, swelling of the feet decreased enough to require a second AFO.

12 months after surgery, customized orthopedic shoes with a stiff sole, cushioned heel, retrocapital rocker, bimalleolar cup (in severe cases with an arthrodesis cup), and smooth, diabetic insole were supplied. At 6 and 12 weeks as well as 6 and 12 months, clinical and radiologic examinations were performed to avoid recurrence and check for skin lesions. Bony consolidation and remineralization were documented with radiographs. The goal of treatment is a plantigrade and stable foot for ambulation. In our opinion, complete consolidation of arthrodesis is not necessary; however, a stable pseudarthrosis at least should be present.

The observed data were entered into a computerized database (Microsoft Excel 2003) and statistically evaluated using IBM SPSS Statistics Software for Windows Release 20.0.0 (2011, Chicago). Categorical variables are expressed as frequency and percentage. Whereas continuous and ordinal variables are presented as mean ± SD. The Marginal-Homogeneity-test was used to compare the difference between two dependent ordinal variables (pre-op. vs. post-op. Activity level). Differences were considered significant at p < 0.05.

## Results

292 feet (282 patients) were included in the study, with 189 male (77.1%) and 93 female (32.9%) patients (Table  1). Polyneuropathy was present in all patients. Most patients suffered from type 2 diabetes (73.4%) and type 1 diabetes (13.1%). 38 patients exhibited polyneuropathy and Charcot neuroarthropathy from other causes (Table  1A, B).

Common secondary diagnoses were retinopathy with an increased falling risk, hypertension, and nephropathy. 26% of type 2 diabetes patients also suffered from peripheral vascular disease (Table 1A, B).

Before surgery, ulcers were present in 184 patients (63%). Ulceration recurred in 67 feet (23%).

All Types of Sanders were included. A combination of Types II and III (Sanders) were most common. The mid-foot was most commonly affected, and a combined involvement was present in nearly all patients (therefore percentage is more than 100%). Type II (76.9%) and III (81.4%, classification of Sanders) were most common. Type I showed the lowest rate of affection (34.1%).

Patients’ activity levels improved significantly (p < 0.001) by 1.01 points with a standard deviation of 0.7 (Table 2).

**Table 2:** **Ability to walk pre**- **and post**-**operative (according to the Hoffer activity level)**

Activity level	Pre-operation	Post-operation	p-value
0	61	3	
1	95	28	
2	84	153	
3	6	76	
Drop out	46	32	
Average level	1.13 (SD 0.81)	2.14 (SD 0.67)	
Total feet	292	292	p < 0.001

Initial amputation was completely avoided. 24 months after surgery, 87 patients (44.6%) were already able to walk in customized orthopedic shoes and 88 (45.3%) in AFOs. Only 6.1% of the patients had to be amputated secondarily due to a failed first surgery and seven patients had died (Table 3).

**Table 3:** **A) Surgery outcome and complications, B) use of orthopedic devices**

Pseudarthrosis	38 (19.6%)
Pin failure/dislocation	3 (1.5%)
Dehiscence	13 (6.7%)
Exostosis	4 (2.1%)
Infection	4 (2.1%)
Ulcer excision	1
Other	5 (2.5%)
Amputation	12 (6.2%)
Death	7 (3.6%)
Customized orthopedic shoes	87 (44.6%)
AFO	88 (45.3%)
Total	194 = 100%
Drop-out	88

Only 19.6% of the cases resulted in pseudarthrosis. Other reasons for revisions were dehiscence (6.7%) and infection (2.1%).

## Discussion

Charcot neuropathy is a severe chronic disease most often due to diabetes mellitus, which remains a challenge for surgeons.

In this largest study investigating long-term outcomes after Charcot foot reconstruction, the use of an external fixator proved to be a safe and cost-effective procedure with few severe complications.

Initial amputations were avoided completely, and the foot was retained in 93.8% of cases overall (Table 3). There is a debate about the cost and effectiveness of foot reconstruction versus transtibial amputation and Gil showed 2013 that the costs of reconstruction of Charcot feet might be similar to transtibial amputation in preliminary data[13]. In our opinion amputation should only be performed, if there is no other choice to save the foot and whenever possible partial amputations of the foot should be performed. As mentioned in the Article of Gil, too, rehabilitation after transtibial amputation and especially problems concerning the stump can be challenging. Usually it takes a long time from surgery until walking after transtibial amputation. We believe that fast recovery after surgery and the ability to walk with full weight bearing is essential for these high-risk patients.

In a prospective clinical study of 26 patients undergoing mid-foot correction using external fixation, Pinzur yielded 24 infection-free and ulceration-free, ambulating patients. One patient died, and one required amputation[14]. This is the only prospective study of Charcot feet identified by our search; however, its scientific value is limited by the small number of patients. In another publication Pinzur described in 2004 the outcome of surgical versus accommodative treatment for Charcot of the midfoot; 63 patients underwent surgery (3 initial amputations) and out of these patients 5 patients (8.5%) had amputation after failure of the salvage surgery[15]. Surgery using a fixateur externe as salvage procedure for osteomyelitis in 178 patients with Charcot Neuroarthropahty was published by Pinzur in 2012; in 95.7% of the patients the limb could be saved, three patients had to be amputated[16].

In our study walking ability improved significantly from activity class 1.13 before surgery to an average of activity class 2.14. The activity class of 1.13 means that most patients could not walk alone (without therapist) at all or only single steps before the intervention. They were mostly depended on help every day and were more or less excluded from social life, as well. Being unable to walk causes high cost for the care and transport of the patients. After surgery patients were able to walk alone inside and sometimes outside (Table 2). Walking ability is extremely important for the cardiopulmonary health of patients, and enables independent living. This is cost-sparing, prevents falls and associated complications, and integrates patients in society. Thus, we deemed this outcome parameter as important and selected it for use in the current study. Further clinical prospective studies are needed to quantify the effect of walking ability to survival rates, hospitalizations, dependence and costs of care for these patients.

Use of the external fixator yielded a high fusion rate, with only 19% resulting in unstable pseudarthrosis requiring revision. The external fixator offers the advantage that all foreign material is removed after six weeks, and thus, cannot fracture or degrade and migrate within the body. Broken remnants are often difficult to remove and can result in internal pressure and ulceration in this high-risk population. Because of the polyneuropathy, patients often do not even recognize shifted hardware; thus, ulceration and infection are dangerous potential complications. As previously mentioned, Charcot neuropathy is a lifelong condition, and this must be considered in planning the procedure.

We saw a high drop-out of 88 patients within 24 months (Table 3). Our university clinic has been a centre for treating Charcot neuroarthropathy since the 1990s, therefore patients are sent to our clinic with an average distance of more than 120 km. That is why many patients are send in critical situations or for surgery but do not usually come regularly as outpatients for controls, if there are no major problems. Another reason is that especially the patients who were able to use a customized orthopedic shoe after 12 months did not come for further consultations, because they did see reason to travel a long distance without major problems.

In most cases, different areas of the foot were affected according to the Sanders classification, most commonly the midfoot (Sanders II and III).

In patients without Diabetes mellitus most often alcohol abuse and rheumatoid arthritis caused polyneuropathy (Table 1B). That is a remarkable result as “Rheumatic Charcot feet” rarely are described. Grear stated in his article that the orthopedic community has to be more aware of the relationship of these two diseases 2013[17]. Charcot feet due to idiopathic neuropathy represent another entity, which just recently had been published and has not been thoroughly investigated[18]. In our opinion, there could be a high number of undiagnosed patients specifically in the early stages of CN in patients without diabetes mellitus, because many physicians combine Charcot neuroathropathy strictly to diabetes mellitus.

The rate of ulceration could be lowered from 184 (63%) to 67 feet with a recurrence/persistance (23%).

Limitations to this study are the high drop out of patients. Further a prospective trial comparing the outcomes of internal and external fixation would give more information.

## Conclusions

Reconstruction of Charcot feet showed to be a save and cost-effective procedure. Severe complications were rare with a low amputation rate. The activity level could be improved significantly by more than 1 level.

## Authors’ original submitted files for images

Below are the links to the authors’ original submitted files for images. Authors’ original file for figure 1

## Abbreviations

CN: Charcot neuroarthropathy
BMI: Body mass index.
