# Supplementary figures and images for: Clinical benefit and improvement of activity level after reconstruction surgery of Charcot feet using external fixation: 24-months results of 292 feet
Source: BMC Musculoskelet Disord. 2014 Nov 22;15:392. doi: 10.1186/1471-2474-15-392 (PMC4289265; doi:10.1186/1471-2474-15-392)

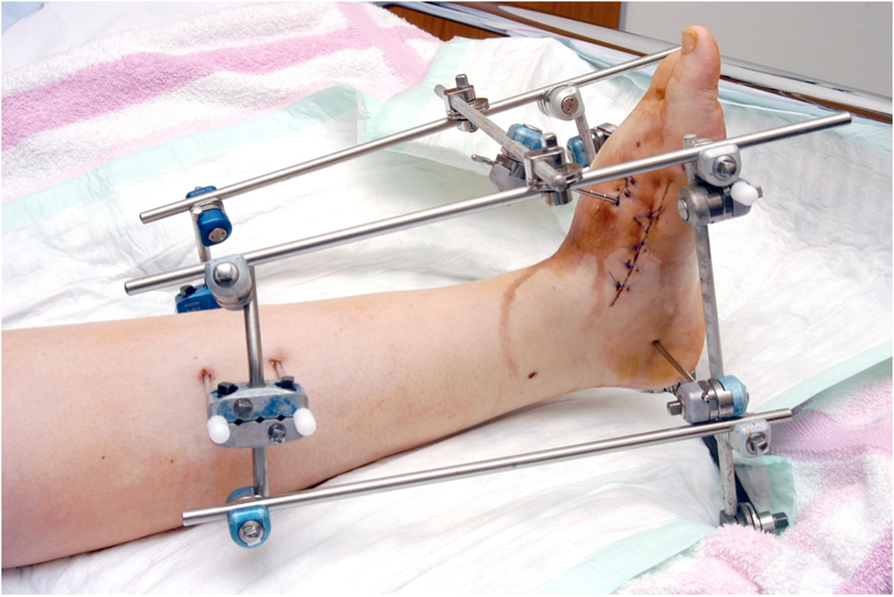

Supplement: Supplementary file 1 — Authors’ original file for figure 1 [file 12891_2014_2365_MOESM1_ESM.tif]
